# Mercury from chlor-alkali plants: measured concentrations in food product sugar

**DOI:** 10.1186/1476-069X-8-2

**Published:** 2009-01-26

**Authors:** Renee Dufault, Blaise LeBlanc, Roseanne Schnoll, Charles Cornett, Laura Schweitzer, David Wallinga, Jane Hightower, Lyn Patrick, Walter J Lukiw

**Affiliations:** 1 United Tribes Technical College, Bismarck, ND, USA; 2 Carl Hayden Bee Research Center, Tucson, AZ, USA; 3 Department of Health and Nutrition Sciences, Brooklyn College of CUNY, Brooklyn, NY, USA; 4 Department of Chemistry and Engineering Physics, University of Wisconsin-Platteville, Platteville, WI, USA; 5 Institute for Agriculture and Trade Policy, Minneapolis, MN, USA; 6 Department of Internal Medicine, California Pacific Medical Center, San Francisco, CA, USA; 7 Contributing Editor, Alternative Medicine Review, Durango, CO, USA; 8 Professor of Neuroscience and Ophthalmology, LSU Neuroscience Center. Louisiana State University Health Sciences Center, New Orleans, LA, USA

## Abstract

Mercury cell chlor-alkali products are used to produce thousands of other products including food ingredients such as citric acid, sodium benzoate, and high fructose corn syrup. High fructose corn syrup is used in food products to enhance shelf life. A pilot study was conducted to determine if high fructose corn syrup contains mercury, a toxic metal historically used as an anti-microbial. High fructose corn syrup samples were collected from three different manufacturers and analyzed for total mercury. The samples were found to contain levels of mercury ranging from below a detection limit of 0.005 to 0.570 micrograms mercury per gram of high fructose corn syrup. Average daily consumption of high fructose corn syrup is about 50 grams per person in the United States. With respect to total mercury exposure, it may be necessary to account for this source of mercury in the diet of children and sensitive populations.

## Background

Chlorine and caustic soda are produced at chlor-alkali plants using mercury cells or the increasingly popular membrane technology that is mercury free and more energy-efficient. Worldwide there are approximately fifty mercury cell chlor-alkali plants in operation [1]. Of those there are eight in the United States (US) [2]. In 2003 the EPA reported in the Federal Register that on average approximately seven tons of mercury were missing from each plant in the year 2000 [3]. These chlor-alkali plants have an average of fifty-six cells, each containing as much as 8,000 pounds of mercury [4] and, every year the chlor-alkali industry reports unaccounted for mercury losses to the EPA [5]. Mercury is a danger to unborn children whose developing brains can be damaged if they are exposed to low dose microgram exposures in the womb [6]. Since mercury is a potent neurological toxin, these unaccounted for mercury losses from the chlor-alkali industry are of concern as they could be a source of exposure for humans, wildlife, and the environment. An Environmental Health Officer (EHO) at the Food and Drug Administration (FDA) conducted an investigation to find the missing mercury in the chlor-alkali industry [7].

## The path of the investigation

An employee of the Environmental Protection Agency (EPA) suggested that the EHO contact the Wisconsin Department of Natural Resources (DNR) for information on Vulcan Chemicals' mercury balance sheet. Vulcan Chemical was the only chemical company to find its missing mercury. Upon request, the Wisconsin DNR provided the EHO with Vulcan Chemical's annual mercury balance sheet that reported their mercury losses in their products for the year that the mercury balance was done. Vulcan Chemical submitted this mercury balance sheet to the Wisconsin DNR in 2003 with their wastewater discharge permit re-issuance application. This information led to the realization that mercury residue may be found in all products produced by the mercury cell chlor-alkali industry. A representative of the Chlorine Institute confirmed in a telephone interview that the amount of mercury residue in mercury cell chlor-alkali products varies, depending on the manufacturing process at each plant. It is found in mercury grade caustic soda according to product specification sheets [8].

According to an archived web page report initially produced by Vulcan Chemicals, mercury grade caustic soda and hydrochloric acid are primarily used by the high fructose corn syrup industry [9]. Following this lead, the EHO conducted an interview with an "organic" producer of high fructose corn syrup (HFCS) in 2004 and was told that the HFCS industry uses both mercury grade caustic soda and membrane grade caustic soda in their manufacturing process to enhance product shelf life. A review of the literature revealed that HFCS is indeed used as a sweetener by food manufacturers to stabilize food products and enhance product shelf life [10]. HFCS is the end product from a corn wet-milling process that involves a number of steps in a product line that yields corn oil, animal feed, starch products, and corn sweeteners. Several chemicals are required to make HFCS, including caustic soda, hydrochloric acid, alpha-amylase, gluco-amylase, isomerase, filter aid, powdered carbon, calcium chloride, and magnesium sulfate [11]. The caustic soda and hydrochloric acid are used throughout the milling process to adjust the pH of the product line. The product line starts with corn and the cornstarch molecule is then converted to different products by various methods that involve acids, bases, sodium hypochlorite and enzymes [12]. Should mercury grade caustic soda, hydrochloric acid, or sodium hypochlorite (derived from mercury grade chor-alkali chemicals) be used in the milling process, it seemed plausible to the EHO that mercury may well end up in the final product – HFCS. A limited screening of HFCS samples for mercury was initiated by the EHO and researchers at NIST found low levels of total mercury. [13].

To determine the extent of total mercury in HFCS products, the EHO then used additional government resources to collect HFCS samples from different manufacturers and collaborate with individuals outside of the federal government to analyze the samples for total mercury content. It should be noted that these activities occurred before the EHO retired in January 2008.

## HFCS sample collection and analytical method

The EHO working under the Office of the FDA Commissioner instructed an investigator in a FDA regional office to collect HFCS samples from different manufacturers. During the week of February 17–24, 2005, the FDA field investigator successfully conducted three separate sampling events, one at each manufacturer. Prior to each sampling event, the FDA field investigator soaked the 20-milliliter (mL) sample vials overnight in a 50 percent (%) nitric acid solution and then rinsed them with distilled water before allowing them to air dry. Per directed assignment from the FDA researcher, the FDA field investigator collected five samples of 42% HFCS and five samples of 55% HFCS from Manufacturer A, five samples of 42% HFCS from Manufacturer B, and five samples of 55% HFCS from Manufacturer C. Each 20 mL sample vial contained approximately 10 mL of HFCS at the end of each of the sampling events. Each sample vial was appropriately labeled with the manufacturers name, % HFCS, date, and the initials of the field investigator. All samples were kept under lock and key prior to being shipped via FEDEX overnight to a laboratory for analyses.

Researchers at the University of Wisconsin-Platteville received the samples from a federal employee with chain-of-custody intact and sub-sampled them for total mercury analysis using NIST Oyster Tissue 1566 b as the standard reference material. The NIST Certificate of Analysis for the Oyster Tissue 1566 b stated that as a standard, it could validate the accuracy of the methods and instruments used to analyze twenty-two different elements including total mercury. All samples, blanks (water and acid matrix), and NIST standard reference material Oyster Tissue 1566 b were analyzed by the following method using Optima Grade Fisher Scientific hydrochloric and nitric acids that were certified to contain less than 0.0001 microgram (μg) mercury per gram (g) reagent. Approximately 1.0 g (to nearest 0.1 milligram) of a HFCS sample, blank, or reference material was accurately weighed into a clean 50 mL XP1500 Plus microwave cell. Approximately 5 mL of nitric acid (Optima Grade Fisher Scientific) was added to the cell. The cell was sealed, and the contents were digested in a high-pressure microwave oven (CEM Mars 5). The resulting solution was allowed to cool before gravimetrically diluting the sample to 50.0 grams (to nearest 0.1 milligram) with 2 Molar (M) hydrochloric acid (Optima Grade Fisher Scientific; 18 MÙ-cm water). Each sample was analyzed within three hours to minimize mercury loss.

A Leeman Labs Hydra AA cold vapor atomic absorption spectrometer (CVAAS) was used for the total mercury analysis. A calibration curve ranging from 10 to 200 picograms mercury/g was constructed using gravimetric dilutions (2 M hydrochloric acid described above) of a primary standard mercury solution (GFS Chemicals). Samples, blanks, and reference materials were introduced along with stannous chloride (GFS Chemicals) reductant at a rate of 5 mL/minute. Each sample and reference material was analyzed in triplicate.

## Results of analyses

Inter-sample blanks displayed no mercury signal above the method detection limit of 0.005 μg mercury/g sample. Mercury recovery of spiked reference materials (GFS Chemicals) averaged 98.8 ± 0.3 %. The results from the total mercury analysis of NIST reference material Oyster Tissue 1566 b (0.036 ± 0.006 μg/g mercury) exhibited good agreement with certified values (0.037 ± 0.001 μg/g mercury). The NIST Oyster Tissue 1566 b analyses were performed prior to samples, between samples, and post-samples with no significant difference (p < 0.05) in the total mercury content between these analyses.

Mercury was detected in nine of the twenty samples analyzed (Table 1). Of ten samples from manufacturer "A", nine were below the 0.005 μg mercury/g sample detection limit with the sole exception being a sample that was 0.012 μg mercury/g HFCS. Of the remaining ten samples from two other manufacturers, two were below the detection limit and the mercury content of the other eight samples ranged from 0.065 μg to 0.570 μg mercury/g HFCS (Table 1).

**Table 1:** total mercury (Hg) in high fructose corn syrup (HFCS) samples

Sample Name	Hg content (μg Hg/g HFCS)		Sample Name	Hg content (μg Hg/g HFCS)
MANUFACTURER A HFCS 42% (sample 1)	< DL (0.005)		MANUFACTURER B HFCS 42% (sample 1)	< DL (0.005)

MANUFACTURER A HFCS 42% (sample 2)	< DL (0.005)		MANUFACTURER B HFCS 42% (sample 2)	< DL (0.005)

MANUFACTURER A HFCS 42% (sample 3)	0.012		MANUFACTURER B HFCS 42% (sample 3)	0.350

MANUFACTURER A HFCS 42% (sample 4)	< DL (0.005)		MANUFACTURER B HFCS 42% (sample 4)	0.390

MANUFACTURER A HFCS 42% (sample 5)	< DL (0.005)		MANUFACTURER B HFCS 42% (sample 5)	0.065

MANUFACTURER A HFCS 55% (sample 1)	< DL (0.005)		MANUFACTURER C HFCS 55% (sample 1)	0.130

MANUFACTURER A HFCS 55% (sample 2)	< DL (0.005)		MANUFACTURER C HFCS 55% (sample 2)	0.400

MANUFACTURER A HFCS 55% (sample 3)	< DL (0.005)		MANUFACTURER C HFCS 55% (sample 3)	0.570

MANUFACTURER A HFCS 55% (sample 4)	< DL (0.005)		MANUFACTURER C HFCS 55% (sample 4)	0.110

MANUFACTURER A HFCS 55% (sample 5)	< DL (0.005)		MANUFACTURER C HFCS 55% (sample 5)	0.240

DL – Detection Limit

## Implications

Mercury was not detected in eleven out of twenty HFCS samples analyzed (detection limit 0.005 μg mercury/g). A single manufacturer produced nine of these eleven samples. These samples were likely manufactured using caustic soda produced by a membrane chlor-alkali plant which does not use mercury in its manufacturing process. Eight of the nine HFCS samples exhibiting mercury levels between 0.065 μg to 0.570 μg mercury/g HFCS were produced by the other two manufacturers. This could indicate the use of mercury grade caustic soda or hydrochloric acid in the manufacturing processes used by these two manufacturers. Such use would account for the mercury in these HFCS products. With key aspects of the HFCS manufacturing process considered proprietary information, we could not confirm the composition of the raw materials used by the individual HFCS manufacturers and the subsequent source of the mercury. While more sophisticated methods produce lower detection limits, the CVAAS method used in these analyses was sufficient as it clearly and reliably demonstrated significant levels of mercury in 45% of the HFCS samples analyzed. Clearly the sample size of this preliminary trial is too small but there was no support to collect additional samples for analyses. When university researchers outside of the government attempted to obtain additional HFCS samples direct from the manufacturer they were unable to get them. However, with 45% of the HFCS samples containing mercury in this small study, it would be prudent and perhaps essential for public health that additional research be conducted by the FDA or some other public health agency to determine if products containing HFCS also contain mercury. In 2004, several member states of the European Union reported finding mercury concentrations in beverages, cereals and bakery ware, and sweeteners [14] – all of which may contain HFCS. FDA does not currently have a mercury surveillance program for food ingredients such as added sugars or preservatives manufactured with mercury grade chlor-alkali products.

The FDA does analyze some foods for mercury through the ongoing surveillance program known as the Total Diet Study (TDS). The TDS, however, does not test all foods for mercury. Mercury is routinely detected by the TDS in fish, liver, and poultry because farmers routinely use fishmeal and/or fish oil as feed for certain livestock to include chickens, swine, dairy cows, and farmed fish. Animals that are fed fishmeal can bioconcentrate monomethyl mercury in protein matrices that are then passed on to the consumer in the fat components of derived foods [15]. A list of the foods that were recently tested for total mercury along with the results of the analyses may be found at the FDA website [16]. In 2003, FDA tested 48 foods for mercury during the TDS and of those only three may have contained HFCS. Average daily US consumption of HFCS for the year 2007 was approximately 49.8 g per person according to the US Department of Agriculture website [17]. High-end consumers of beverages sweetened with HFCS could easily be ingesting more HFCS than the average person. Results of a recent study of dietary fructose consumption among US children and adults indicate that fructose consumption by Americans represents ten percent (10%) of calories consumed in a 24-hour period [18]. Seventy four percent (74%) of this fructose came from foods and beverages other than fruits and vegetables.

With respect to product labeling, FDA requires food manufacturers to list on the food product label ingredients in descending order of weight from most to least [19]. For example, HFCS is commonly listed as the first ingredient in chocolate syrup on the product label, therefore all that can be known is that of all the ingredients in chocolate syrup, there is more HFCS in the product than any other ingredient. Product labels listing HFCS as a first or second ingredient may contain detectable levels of mercury if the HFCS was manufactured with mercury grade chlor-alkali chemicals. As part of the review process for this article, the authors contacted manufacturers for more information on the % concentration of HFCS in their products and the common response back from manufacturers was that this information is proprietary. With the reported average daily consumption of 49.8 g HFCS per person, however, and our finding of mercury in the range of 0.00 to 0.570 μg mercury/g HFCS, we can estimate that the potential average daily total mercury exposure from HFCS could range from zero to 28.4 μg mercury. This range can be compared to the range of total mercury exposure from dental amalgam in children reported by Health Canada [20]. In the report issued by Canada, daily estimates of total mercury exposure from dental amalgam in children ages 3–19 ranged on average from 0.79 to 1.91 μg mercury. Canada and other countries do not recommend the use of mercury amalgam in pregnant women or children.

Current international food processing standards allow 1.0 μg mercury/g caustic soda [21,22] and there is no standard for mercury in food grade hydrochloric acid. Both of these chemicals may be used to make HFCS. The FDA has approved HFCS for use as an added sugar in food products but a review of food product labels reveals that it is often added to a product in addition to sugar presumably to enhance product shelf life. Regardless of its intended use, it is imperative that public health officials evaluate this potential source of mercury exposure, as HFCS is presently ubiquitous in processed foods and therefore significantly consumed by people all over the world.

Mercury in any form – either as water-soluble inorganic salt, a lipid-soluble organic mercury compound, or as metallic mercury- is an extremely potent neurological toxin [23]. Organic mercury compounds such as methylmercury that are fat-soluble and readily cross the blood brain barrier are especially damaging to developing nervous tissues [24,25]. For example, prenatal exposure as low as 10 mg/kg methylmercury, as measured in maternal hair growing during pregnancy, may adversely affect the development of the fetal brain [25,26]. Confounding associations and concerns with various stages of brain development related to cumulative early life exposure to mercury include the following sources of mercury: maternal fish consumption during pregnancy, the thimerosal (sodium ethylmercurithiosalicylate, approximately 49% mercury weight) content of certain vaccines and dental amalgam [27].

Mercury regulation varies from country to country. While the US government only regulates methylmercury in fish, several other governments regulate all forms of mercury in all foodstuffs. In the US, the current action level of 1 μg methylmercury/g fish or seafood was set in 1977 during court proceedings of the United States of American v. Anderson Seafoods, Inc. [28]. The data used to determine the action level in fish came from a poisoning incident that occurred in Iraq under Saddam Hussein's regime in 1971–1972. There was not a chain of custody for the specimens taken from the victims of that poisoning that were tested by World Health Organization or American researchers, and an appropriate epidemiological study was not undertaken [29]. Further risk assessment for methylmercury has been conducted using human data from the massive episodes of mercury poisoning in the tragic Minimata Bay incident in Japan, as well as from large scale epidemiological studies concerning childhood neurodevelopment and neurotoxicity in relation to fetal exposure in various fish eating communities around the world [24,25]. There has never been a blinded, placebo, controlled study published giving humans mercury or methylmercury, nor would this kind of study be ethically considerable. Quantitative information on long-term effects of inorganic mercury compounds on humans does not exist [30]. Inorganic mercury compounds react with DNA and are clastogenic [30]. Because the mechanisms of these reactions remain unknown, it is currently impossible to establish a no adverse-effect-level for mercury in humans. Sensitive populations such as neonates lacking the ability to efficiently excrete mercury or individuals that retain mercury in their body due to impairments in detoxification pathways may not be protected by any exposure limit. The implications for mercury in ingested HFCS are not known and clearly more epidemiological and neurotoxicological studies are required.

## Conclusion

An EHO at the FDA conducted an investigation of the chlor-alkali industry in 2004 and found mercury residue in all of the mercury cell chlor-alkali products including caustic soda, chlorine, potassium hydroxide, and hydrochloric acid. Mercury is widely accepted to be a neurotoxic heavy metal [23]. The American Academy of Pediatrics has recommended that minimizing any form of mercury exposure is essential for optimal child health and nervous system development [6]. Current international food processing standards allow 1.0 μg mercury/g caustic soda [21,22] and there is no standard for mercury in food grade hydrochloric acid. Both of these chemicals may be used to make HFCS. Mercury contamination of food products as a result of the use of mercury contaminated HFCS seems like a very real possibility. With daily per capita consumption of HFCS in the US averaging about 50 grams and daily mercury intakes from HFCS ranging up to 28 μg, this potential source of mercury may exceed other major sources of mercury especially in high-end consumers of beverages sweetened with HFCS. Food products that contain a significant amount of HFCS should be tested for mercury contamination in the end product and the public should be informed of any detections. Clearly, more research is needed to determine the extent of mercury exposure in children from mercury contaminated HFCS in food products.

## Abbreviations

AA: Atomic Absorption; CEM: Corporation that makes microwave accelerated reaction systems; CVAAS: Cold Vapor Atomic Absorption Spectrometer; DNR: Department of Natural Resources; EHO: Environmental Health Officer; EPA: Environmental Protection Agency; FDA: Food and Drug Administration; GFS: Grade Fisher Scientific; HFCS: High Fructose Corn Syrup; M: Molar; MARS: Microwave Accelerated Reaction System; NIST: National Institute of Standards and Technology; TDS: Total Diet Study; US: United States; XPI: Cross Polar Isolation

## Competing interests

The authors declare that they have no competing interests.

## Authors' contributions

RD carried out the environmental investigation, conceived the study, acquired the samples for analyses, and was involved in drafting the manuscript. BL provided assistance in the study design and was involved in drafting the manuscript. CC and LS analyzed the samples, acquired the data and were involved in drafting the manuscript. RS, LP, JH, DW and WJL were all instrumental in drafting the manuscript and revising it critically for intellectual content. All authors read and approved the final manuscript.
